# Supplementary material for: A Pilot Study of Integrated Digital Tools at a School-Based Health Center Using the RE-AIM Framework
Source: Healthcare (Basel). 2025 Jul 29;13(15):1839. doi: 10.3390/healthcare13151839 (PMC12346205; doi:10.3390/healthcare13151839)
Supplement: Supplementary file 1 [file healthcare-13-01839-s001.zip › healthcare-3636336-supplementary.pdf]

Table S1: Characteristics of the Adolescent Patients who used the app

	RAAPS N=21	Health-E You N=53
Age (mean/SD)	15.62	15.98
Race/ethnicity		
Latine/Hispanic	100%	80.39%
Black/African American	0%	3.92%
White	0%	3.92%
Asian	0%	1.96%
Native American/Alaskan Native	0%	1.96%
Sex at Birth		
Female	71.43%	71.70%
Male	28.57%	28.30%
Gender Identity		
Female	66.67%	67.92%
Male	28.57%	28.30%
Gender Fluid/Gender nonbinary	4.76%	1.89%
Transgender	0%	1.89%
Sexual intercourse (Female Sex Only)		
Yes	61.90%	59.46%
No	38.10%	40.54%
Contraceptive use prior to using app		
Birth Control Pills	n/a	7.69%
Depot Shot	n/a	0.00%
Male Condom	n/a	53.85%
Implant	n/a	15.38%
Pull-out	n/a	23.08%
App Recommendations		
LARC (IUD or Implant)	n/a	76.92%
Depot Shot	n/a	7.69%
Ring/patch	n/a	15.38%
Method interested in Using		
Not ready to select a method	n/a	57.14%
Intra-uterine device (IUD)	n/a	7.14%
Depot Shot	n/a	14.29%
Birth Control Pills	n/a	14.29%
Patch	n/a	7.14%
Time to complete in minutes: Mean (SD)	5.52 (3.51)	6.73 (1.80)
